# A Framework for Development of Useful Metabolomic Biomarkers and Their Effective Knowledge Translation

**DOI:** 10.3390/metabo8040059

**Published:** 2018-09-30

**Authors:** Calena R. Marchand, Farshad Farshidfar, Jodi Rattner, Oliver F. Bathe

**Affiliations:** 1 Faculty of Engineering, University of Waterloo, Waterloo, ON N2L 3G1, Canada; cmarchand@edu.uwaterloo.ca; 2 Department of Oncology, University of Calgary, Calgary, AB T2N 1N4, Canada; farshidf@ucalgary.ca; 3 Arnie Charbonneau Cancer Research Institute, University of Calgary, Calgary, AB T2N 1N4, Canada; jodi.rattner1@ucalgary.ca; 4 Department of Surgery, University of Calgary, Calgary, AB T2N 1N4, Canada

**Keywords:** metabolomics, biomarkers, knowledge translation

## Abstract

Despite the significant advantages of metabolomic biomarkers, no diagnostic tests based on metabolomics have been introduced to clinical use. There are many reasons for this, centered around substantial obstacles in developing clinically useful metabolomic biomarkers. Most significant is the need for interdisciplinary teams with expertise in metabolomics, analysis of complex clinical and metabolomic data, and clinical care. Importantly, the clinical need must precede biomarker discovery, and the experimental design for discovery and validation must reflect the purpose of the biomarker. Standard operating procedures for procuring and handling samples must be developed from the beginning, to ensure experimental integrity. Assay design is another challenge, as there is not much precedent informing this. Another obstacle is that it is not yet clear how to protect any intellectual property related to metabolomic biomarkers. Viewing a metabolomic biomarker as a natural phenomenon would inhibit patent protection and potentially stifle commercial interest. However, demonstrating that a metabolomic biomarker is actually a derivative of a natural phenomenon that requires innovation would enhance investment in this field. Finally, effective knowledge translation strategies must be implemented, which will require engagement with end users (clinicians and lab physicians), patient advocate groups, policy makers, and payer organizations. Addressing each of these issues comprises the framework for introducing a metabolomic biomarker to practice.

## 1. Introduction

A “biomarker”, as defined in 1998 by the National Institutes of Health Biomarker’s Definitions Working Group, is “a characteristic that is objectively measured and evaluated as an indicator of normal biological processes, pathogenic processes, or pharmacologic responses to a therapeutic intervention” [1]. Biomarkers are by definition objective and quantifiable representations of biological processes. The majority of biomarkers currently in clinical use are based on genomics, transcriptomics, and proteomics, and more recent biomarker developments have been based on multiplexed measurements. Genomics utilizes information derived from the genome to aid in medical decision making, for instance, to predict certain drug responses [2]. Examples of clinical applications using transcriptomics include the utilization of RNA-sequencing data to predict response to various treatments or suggesting other “omics” platforms that would be suitable when designing an assay [3,4]. Proteomics have been used to determine proteins that serve as biomarkers for early cancer diagnosis and disease progression [5,6]. Metabolomics offers an advantage over the other “omics”, as it can monitor biochemical activity during cellular metabolism on multiple analytical platforms [7,8]. Despite the fact that there has been a significant growth and maturation of the field of metabolomics over the last 10–15 years, few metabolomic biomarkers are in common use today and currently, no metabolomics tests have 510 k clearance by the U.S. Food and Drug Administration (FDA). There is only one biomarker test available that utilizes genomic sequencing for oncology that was given FDA premarket approval [9].

Metabolomics focuses on the presence of small molecules in various complex matrices including blood, urine, saliva, and other human tissues. The metabolome represents a functional portrait of the state of health of any individual; it changes rapidly and measurably with changes in state. Moreover, because various metabolites are co-related (either physiologically or stoichiometrically), the exact pattern of abundance of metabolites may reflect a unique pathophysiological state. Importantly, the metabolome may be a much closer molecular representation of phenotype and present state of health or function than any biomarker that is based on the genome, transcriptome, or proteome. It is the dynamic nature of the metabolome, as well as its close relationship with phenotype, that has stirred substantial interest in the research community. The temporal nature of the metabolome makes it particularly intriguing, as it is a real time reflection of a patient’s state of health.

A metabolomic biomarker is very different from a protein biomarker and transcriptomic biomarkers, in view of the close relationship between individual metabolites. The factors measured in other “omics” technologies are independent, although there may be patterns of abundance that reflect a disease state. A metabolomic biomarker, however, is not just a string of changes in individual metabolites. Rather, it is comprised of groups of co-related metabolites that change in concert; it is a meta-biomarker. This is very different from a biomarker consisting of individual metabolites that independently vary with disease; the interdependence of metabolites results in a disease signature. The “signature” may change as a disease process evolves. For example, in a patient with chronic obstructive pulmonary disease, there may be progressive emphysematous changes in the lungs which produce a continuum of changes in the metabolome. Then, another set of changes may appear as cancer evolves. We and others have observed this with cancers at various stages [10,11]. Importantly, because a metabolomic biomarker is a meta-biomarker, a random change in a single metabolite will not provide a false signal. A metabolomic biomarker is therefore a powerful means of monitoring changes in a person’s condition over time.

Why then are metabolomic biomarkers not emerging for use in the clinic? The purpose of this paper is to identify obstacles to metabolomic assay development and to propose a framework for more efficient assay development. In addition to delineating the essentials of assay development, a roadmap for effective knowledge translation is described. Knowledge translation describes moving research to the public domain, including to the clinic as well as to commercialization. We speculate that adherence to these principles will accelerate effective introduction of metabolomic biomarkers to clinical use.

### 1.1. Identifying the Clinical Application: the Prime Objective

Biomarker work must be driven by the clinical need, and the experimental methods (including sample procurement from the properly designed clinical cohorts and controls) must be dictated by the biomarker purpose. Unfortunately, many metabolomic biomarker studies are done based on samples that are already available, and the biomarkers derived are purposed after the fact. Prospective study design and collection represents an optimal approach, although it is not always pragmatic.

Metabolomic biomarker studies require interdisciplinary collaboration. Most experts in metabolomics have little insight on the clinical conditions of sample collection. Many also have relatively little appreciation of the clinical need for specific biomarkers as well as how they dictate management. At the same time, most clinicians have very little understanding of the technical nuances of metabolomic studies as well as the biochemical interdependencies of metabolites. For some study designs, new statistical approaches must be explored. The natural solution is an interdisciplinary collaboration that is both respectful and supportive.

Once the team has decided on the clinical need for a biomarker, then development can begin, now focusing on more technical factors. Close attention to those technical factors, in addition to staying true to the original purpose of the clinical biomarker, should greatly facilitate the legal and regulatory aspects of biomarker development.

### 1.2. The Biomarker Development Process: Setting the Stage for Effective Knowledge Translation

Frequently, metabolomic biomarkers from convenient samples lack a definite end utility [12]. Differentially abundant metabolites are identified, and the investigator asserts that this represents a clinically useful biomarker. The process needs to be reversed. The purpose of the biomarker must be defined *a priori*, and the experimental design for identification of the biomarker must follow accordingly. A suitable cohort with the disease condition or pathophysiological perturbation of interest must be available. With metabolomic biomarkers in particular, it is vital that the biomarker is tested in a population that is representative of the population for which the test is designed: ethnicity and country of origin should be considered. A control group that is relevant to the biomarker purpose must also be identified. A disease-free control group may not always be relevant. For example, a disease-free control group would not be relevant if one were developing a biomarker that is used to distinguish benign and malignant tumors. Importantly, it is critical that the conditions of collection in the test and control groups must be exactly identical. Confounders such as sex and age should always be matched; other confounders that appear systematically should also be controlled [13,14,15,16,17]. Table 1 describes potential confounding factors that should be considered in the experimental design, during discovery and validation of a metabolomic biomarker.

In addition to testing a discovery or training set (to characterize a biomarker), validation is essential. Internal validation presents an estimate about what can be extracted from the assay based on the research setting and design. An internal validation set should be collected from the same population and under the same analytical conditions. This set is required to determine if the proposed biomarker can distinguish the disease state in a cohort that is similar to the discovery cohort. At this phase of validation, it is possible to optimize the biomarker (for example, by including or excluding certain metabolites) or the testing process. If internal validation demonstrates that the biomarker has promise as a classifier, then it should be tested on an external validation set [32]. The external validation set should have the same characteristics as the initial test set. Such characteristics that should be considered include test environment, sample preparation and processing, and patient population features. Patients should be representative of the population most susceptible to the disease, taking into consideration factors such as sex and age. For instance, an age of 50+ years would be suitable for external validation of a colon cancer biomarker [33]. The patient population should also have the same inclusion and exclusion criteria, and collection and processing procedures should resemble what was done in the initial test set. Finally, the biomarker must be tested in a separate prospectively collected cohort. Ideally, this cohort will be assembled by a multi-institutional consortium, employing a standardized testing protocol. Moreover, it is critical that the disease prevalence matches what would be seen in a real test situation. For example, if a biomarker is being developed for disease screening, then its performance must be evaluated in a cohort where the disease prevalence is as low as what would be seen in a real screening population. This development pathway reflects what has been described as the development pathway for a biomarker designed for the purpose of early detection of disease by the Early Detection Research Network (EDRN) [34,35].

## 2. Sample Considerations

Metabolomic studies can be performed on any biofluid, including serum, plasma, urine, cerebrospinal fluid, or tissue extracts [22,36]. The sample type selected should be the most appropriate for addressing the clinical need, taking into consideration that convenient and minimally invasive tests are most likely to be widely accepted. While the clinical purpose of a biomarker is the primary determinant of the choice of biofluid, ignoring the importance of convenience will limit clinical uptake. For example, blood is more convenient than a stool sample, and patients may not be compliant with any test based on a biomarker derived from a stool sample. Tests involving samples that are collected using a more invasive procedure may also be problematic. For instance, tissue obtained by biopsy (especially of sensitive regions such as the brain or deep tissues) is not as easily procured as blood or urine. Therefore, if a test can be adapted using a biofluid that is easily accessed and that would be acceptable to patients, that is always preferable.

Because the metabolomic profile may be biased by environmental and dietary factors, those potentially confounding factors should be controlled if possible. A simple maneuver to control for some of that effect is to ensure that samples are collected after a period of fasting. With the need for fasting, patient compliance does diminish. However, fasting for at least 12 h before blood/urine samples are collected is highly recommended to reduce any dietary effects that are otherwise difficult to control [21]. It has been shown that fasting overnight can significantly impact the concentration of metabolites found in urine [37]. Some biofluids such as semen and blister fluid may be less affected by a shorter period of fasting [21].

There is potential for introducing confounding factors if blood samples are analyzed without purposeful introduction of standardized operating procedures (SOPs). Blood obtained by arterial puncture could be expected to differ in its composition from blood obtained by venipuncture. Similarly, serum (which is devoid of clotting factors such as fibrinogen) has different metabolite concentrations than plasma (where the cell free component of blood has been treated with an anticoagulant) [23]. Degradation is minimized if the sample is flash frozen in liquid nitrogen and then stored at −80 °C [38]. Significant changes in metabolite concentrations appear in samples stored at room temperature after 24 h; transferring samples on cool packs will reduce the extent of changes [26,38,39].

Researchers should avoid excessive freeze-thaw cycles [39], and this can be done by anticipating the amount of sample required for analysis and making multiple smaller aliquots after procurement. The effect of freeze-thaw cycles on the metabolite composition of samples is controversial. Studies have shown that metabolites in each chemical class are minimally affected by 1–4 freeze-thaw cycles [26,28,29,30]. While the specific effects of multiple freeze-thaw cycles have not been defined, given the possibility that even some metabolites may be more sensitive, and because metabolomic biomarkers are defined by the relative concentrations of metabolites to each other, avoiding excessive freeze-thaw cycles still constitutes a good practice.

In general, relevant SOPs must be designed and followed. SOPs will improve the chances of producing reliable and reproducible data [21]. Ideally, SOPS should be formulated early, beginning when samples are collected and stored. The principles embodied by Good Laboratory Practice (GLP) will ensure the generation of high quality and reliable test data; GLP is essential in studies leading to commercialization or clinical applications, and any testing facility implementing a biomarker test that is in clinical use must follow GLP [40,41]. A series of general recommendations regarding standardization of methods for developing clinically relevant biomarkers has been presented by the Consortium of the American Association for Cancer Researchers (AACR), the FDA, and the National Cancer Institute (NCI) [42]. These recommendations include recommendations specific to metabolomic studies.

## 3. Selection of Analytical Platform

One substantial problem in the field is that there are multiple analytical platforms that are capable of surveying the metabolome. Many options can be attractive to researchers, however, there can be large variation between instruments. Certain analytical tools are advantageous in some areas while lack certain important capabilities. For instance, NMR is highly sensitive, yet is very expensive and requires a specialized facility. LC-MS will detect a wider range of metabolites, but comes at the cost of a higher signal-to-noise ratio, making it more difficult to create a stable metabolomic signature. GC-MS is sensitive and capable of a comprehensive analysis of the metabolome. It has a small footprint, and is therefore easily applicable in a clinical lab. However, samples must be derivatized and therefore the measured compounds may not entirely reflect the original concentrations of individual metabolites. The advantages and disadvantages of each of these platforms are summarized in Table 2. Typically, for pragmatic reasons, metabolomic biomarkers are discovered and validated using whatever instrumentation is available. There are some machines that are not well suited to a clinical laboratory with restricted work space and the need for high sample throughput. For example, NMR spectroscopy requires a special facility; and Fourier transform mass spectrometry has slow acquisition rates. The ideal analytical platform for a clinical laboratory is compact, capable of high throughput sample analysis, sufficiently sensitive with high resolution to reliably detect the compounds comprising the metabolomic biomarker, and simple to use and maintain.

In some instances, clinical laboratories must consider repurposing instruments already available at the facility. This may require the adaptation of a metabolomic biomarker developed on one analytical platform (e.g., NMR spectroscopy), then testing for the same compounds on a machine that is available (e.g., LC-MS). The availability of a well-designed assay with appropriate internal standards and software to simplify the biomarker detection process will facilitate that transition.

## 4. Assay Design

The general processes for developing a clinical assay have been well described [36]. However, there is little information describing assays based on metabolomics, and the vast majority of metabolomic biomarkers reported in the literature are based on semi-quantitative methods. This is a significant obstacle to the wider adoption of metabolomics as a platform for biomarker development. An ideal assay would be fast, convenient, inexpensive, accurate, and reliable. The capability to reliably quantify the metabolites that comprise a biomarker is essential.

In order to quantify metabolites on any analytical platform based on mass spectrometry, internal standards are required. Internal standards also reduce measurement variation that is introduced by sample preparation, ionization efficiency, and chromatographic retention [31], enhancing the predictive performance of the biomarker. The most reliable and efficient internal standards are comprised of metabolites labeled with heavy isotopes such as deuterium (^2^H). ^13^C or ^15^N are alternative labels. In principle, internal standards must be easily identifiable by their mass-to-charge ratio and distinct from metabolites that are normally abundant in the biological samples [23]. The assay design should facilitate quantification of all of the metabolites comprising the biomarker. It would be cost prohibitive to include internal standards that match all of the metabolites, even in the most parsimonious of metabolomic biomarkers. Therefore, an alternative approach is to include internal standards that cover the range of chemical classes of metabolites in the biomarker, and that are representative of various retention times. Concentrations of internal standards must be in the linear range of measurement of the analytical instrument. Finally, in the assay procedure, internal standards must be added to samples at the beginning of the preparation process to ensure that they accurately reflect the extraction and derivatization reactions that occur in intrinsic compounds [23].

Any assay must include quality control (QC) samples to distinguish analytical variations (including drift and other technical aberrations) from biological variations. QC samples are also useful to monitor method validity and the performance of the method [23]. QC samples should be comprised of the biofluid being analyzed in test samples, spiked with internal controls at the same concentrations as in test samples. In addition, QC samples should be processed identically to test samples [31], and they should be regularly distributed throughout the batch of samples, to monitor for variations throughout the length of the run.

Once the assay design has been completed, it is essential to re-test the biomarker to ensure that it still reliably identifies the target population that it is meant to identify. This involves testing the biomarker in an adequately powered external validation cohort. If the validation study design involves just testing the biomarker by itself, then the study must have the statistical power to test for an effect size. If the biomarker is compared to another diagnostic test, then statistical power to determine which test has the best diagnostic capability is required. At the end, what is required is to derive a precise estimate of the assay’s performance characteristics include sensitivity, specificity, positive predictive value, negative predictive value, and accuracy. This involves accurately plotting a receiver operating characteristic curve. Hajian-Tilaki has published an excellent review of the considerations involved in estimating sample size for this purpose [52].

Finally, if commercialization is the goal, it is essential to clearly document the processes employed, from initial design to construction of the final diagnostic test [53]. Specifically, this includes minimum procedural requirements to address regulatory organizations’ concerns throughout the approval process [54]. A set of standards for metabolomic assay systems has been created by the Coordination of Standards in Metabolomics (COSMOS) in 2007, to standardize main procedures across metabolomic studies [55]. These procedures allow for the sharing of reliable and reproducible data across a variety of metabolomic studies and also ensure that the metabolites of significance have verified identities and are sufficiently described [56].

## 5. Patent Protection and Regulatory Approval

Patent approval for metabolomic biomarkers has proven to be a challenge due to barriers in the approval process. One difficulty is that metabolites are naturally-occurring compounds. Following the ruling of the Supreme Court of the United States in the case of Mayo Collaborative Services v. Prometheus Laboratories, Inc., metabolomic patents have been viewed as unenforceable. Briefly, the case involved a dispute over whether a method to titrate the dosage of thiopurines for autoimmune disorders could be patented. Individuals’ metabolism of thiopurine drugs varies widely. A method was described to measure the metabolites of thiopurines (6-thioguanine), allowing titration of the drug dosage based on the levels of 6-thioguanine in the blood. The Supreme Court stated “To put the matter more precisely, do the patent claims add enough to their statements of the correlations to allow the processes they describe to qualify as patent-eligible processes that apply natural laws? We believe that the answer to this question is no.” [57].

This case (which pertains to a simplistic assay of a single metabolite) does not mark the end of metabolomic biomarkers, however. Metabolomic biomarkers (comprised of multiple co-related metabolites) are meta-biomarkers. They do not rely on quantification of a single metabolite; rather, they consist of defining patterns of abundance of multiple biomarkers. The biomarker is a mathematical derivative describing the inter-relationship of groups of metabolites that requires innovation; it is a mathematical algorithm that requires the purposeful inclusion and exclusion of specific metabolites to function; and it is not obvious.

Another obstacle to patent protection is the existence of “prior art”. Any publications and patents that describe the differential abundance of single metabolites or groups of metabolites that partially comprise the metabolomic biomarker can be construed as lacking novelty. Again, this is where the concept of a meta-biomarker may be instructive, as the metabolomic biomarker can only be expected to function optimally if all metabolites constituting the algorithm are measured together.

If metabolomic biomarkers are ultimately deemed as non-patentable, then the inability to protect the intellectual property (IP) will stifle development and inhibit investment. Investors may be reluctant to sink funding into any technology where the IP cannot be protected. This represents a potential obstacle for reducing any metabolomic biomarker to clinical practice. Even if metabolomic biomarkers cannot be patented, there may be work-arounds to retain commercial viability. Proprietary diagnostic algorithms could be catalogued, and developers could protect those algorithms. The disadvantage of that situation is that there may emerge a reluctance by researchers to publish the details of their biomarker efforts for fear of other developers stealing those data for the purpose of reducing their own development time.

In addition to IP protection, regulatory approval could represent a barrier to adapting a metabolomic biomarker for clinical use. While it is not essential that a test receives regulatory approval, if the intent is to make the test widely applicable as a standard of care, then FDA approval is essential. Insurance companies and health care constituencies would expect FDA approval prior to widely implementing any test, as it implies that a certain level of performance has been demonstrated and significant evidence for its applicability has been presented. As with any diagnostic test that is being developed for FDA approval, the process is complicated. The FDA has strived to minimize this difficulty. In 2004, the FDA introduced the “Critical Path Initiative” (CPI) to improve the development of drug and medical device treatments [58,59]. The CPI describes assay design considerations, standard clinical trial endpoints, as well as features of biomarkers that will create more effective and efficient treatment to benefit patients, including clinical utility [59].

Currently, most commercial metabolomic assays are performed in highly-controlled research labs holding a CLIA certificate (42 CFR §263a(a)) [60]. Tests in this format are referred to as Laboratory Developed Tests (LDTs).

While the FDA only regulates tests that are sold as kits at this time, the FDA has signaled that it has plans to regulate all LDTs as well as genomic tests, to account for the changing market and increasing complexity [61]. In October 2014, the FDA published notice of Draft Guidance on regulation of LDTs in “Framework for Regulatory Oversight of Laboratory Developed Tests (LDTs)”. At the time of this writing, this document is still being reviewed in U.S. Congress [62], and final guidance for LDTs has not been provided by the FDA.

## 6. Knowledge Translation: Engagement of the Stakeholders

**Figure 1:**
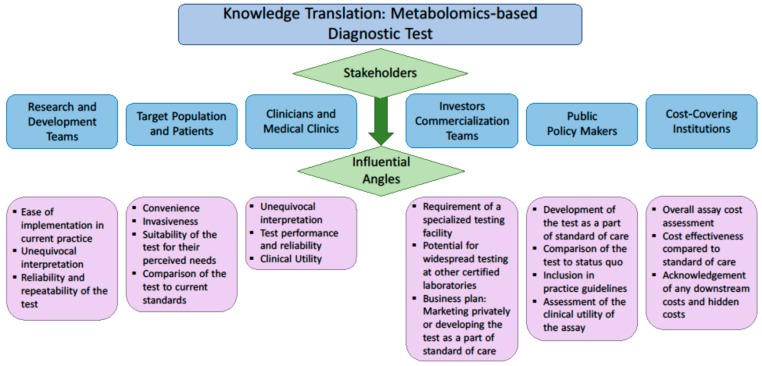
Factors of interest to various stakeholders when evaluating the implementation of a new diagnostic assay.

The demand for molecular diagnostic tests is driven by clinicians’ needs and payers’ interest [63]. Payers include public or private health systems and insurance companies, as well as private consumers. The most important feature of any test for clinicians is the clinical utility, which describes the capability of any test to affect clinical management. To a laboratory physician, the test must be easily implemented in the limited space available in a clinical lab. To the payer, in addition to clinical utility, additional economic features are important. The cost effectiveness of the biomarker must be compared to standard of care; and other economic advantages and disadvantages must also be considered. To the patient, a test must be convenient and minimally invasive, and the information provided by a test must have overt value. Given the diverse drivers and considerations for each variety of stakeholder, to effectively translate a biomarker discovery to practice, it is essential to consult those stakeholders on their perceived value of the test and their understanding of the advantages it may provide. These factors considered by each group of stakeholders are described in Figure 1.

## 7. Conclusions

There are several challenges related to bringing a biomarker from discovery to clinical use, which includes marketing a test. The biomarker need should precede discovery efforts and validation. Excellent experimental design at the beginning will enhance the probability that the biomarker will perform as it is designed. An assessment of clinical utility and a full economic analysis are integral to the more advanced stages of test development. Finally, to effectively translate biomarker discoveries to clinical use, it is essential to have a meaningful consultation with all stakeholders.

## Figures and Tables

**Table 1:** Potential confounding factors that must be considered in the development of a reproducible metabolomic biomarker, in the discovery and validation phase.

Important Factors for Consideration	Conditions and Explanation	Reference Study
**Experimental Conditions**		
Choice of methodology (technology)	Choice of instrumentation should take into consideration the class of metabolites and biological functions of interest.	Putri et al. [18]
	Analysis batches should be balanced with comparator groups, taking into account potential confounders. Corrections must be made for batch variation.	Dunn et al. [19]
Sample preparation	Chemical derivatizations made during the preparation procedure should be designed to specifically react with the target chemical structure and cause minimum alterations to the unintended sites.	Kvitvang et al. [20]
**Patient Factors**		
Diet and drug interactions	Diet and drug exposure may affect metabolite quantities. Therefore, ideally, blood samples should be taken from the patient after 8–12 h of fasting.	Emwas et al. [21]
Physical activity	To avoid the metabolite fluctuation as a result of physical activity, no physical exercise should be done before sample extraction, unless that is a component of the biomarker.	Emwas et al. [21]
Age	Disease state and controls should be age matched.	Ishikawa et al. [16]
Sex	Sex composition of study cohorts should be considered when designing a balanced and unbiased study.	Krumsiek et al. [13]
**Sample Processing**		
Processing time	Tissue dissection should be carried out immediately to minimize sample degradation. Sample collection and processing standardized operating procedures (SOPs) are essential.	Emwas et al. [21]
Processing reagents	The type of coagulant used to create serum samples from plasma might have an effect on the ionization process in analysis.	Qiu et al. [22] Lind et al. [23]
Sample storage and preservation	Samples should be stored at –80 °C to minimize changes in metabolite concentrations.	Emwas et al. [21]
	While some metabolites may be altered at –20 °C, valid metabolomic signatures can be derived as long as comparator groups are stored at the same temperature.	West- Nielson et al. [24] Rai et al. [25]
	Keeping samples on cool packs during processing will reduce degradation as compared to processing at room temperature.	Anton et al. [26]
	Time from sample collection to freezing should be minimized to prevent degradation of some classes of metabolites, including amino acids and biogenic amines.	Breier et al. [27]
	Processing samples shortly after collection is optimal. Samples from different years of collection will have different metabolomic profiles, and this will need to be considered in the batch design as well as the final analysis.	Lind et al. [23]
**Sample Storage**		
Freeze/Thaw cycles	Minimizing freeze/thaw cycles will result in less compositional changes. 1–4 freeze/thaw cycles cause little variation but 5+ cycles cause significant variation.	Anton et al. [26] Kronenberg et al. [28] Zivkovic et al. [29] Yin et al. [30]
**Quality Assurance**		
Quality Control samples	Quality Control (QC) samples are analyzed at the same time as unknown samples to minimize standard error (SE) by accounting for biological or analytical variations.	Phinney et al. [31]
Internal standards	Internal Standards (IS) are used to validate the identity and to quantify metabolites, and should be added to each sample at the beginning of the preparation process.	Phinney et al. [31] Lind et al. [23]

**Table 2:** Features of analytical platform for metabolomics that should be considered when devising a clinical assay based on metabolomics.

Type of Analytical Platform	Advantages	Disadvantages	References
Nuclear Magnetic Resonance (NMR)	Non-destructive (samples can be recovered for further analysis). Relatively easy sample preparation. Good for complex mixtures (respective intensities of metabolites are not affected by others). NMR Spectra can easily be validated with 2D Spectroscopy methods (TOCSY or STOCSY).	Higher detection threshold than Mass Spectrometry. Requires a large, expensive, and specialized facility (impractical for most clinical labs).	Putri et al. [18]
Mass Spectrometry			
Gas Chromatography-Mass Spectrometry (GC-MS)	Detects low molecular weight compounds (e.g., amino acids, sugars, etc.). Compact machine (smaller footprint for clinical laboratory). High peak capacity to cover a wide range of concentrations. Utilizes Electron Ionization (EI) to increase ionization efficiency. Normalized retention indices for each compound. Reliable standard operating procedures (SOPs).	Samples must be volatile in order to pass through the machine (requires compound derivatization). Derivatization alters compounds’ chemical structure which could misrepresent desired compound after fragmentation in MS. Higher molecular weight compounds are insufficiently volatile.	Putri et al. [18] Nordström et al. [43]
Liquid Chromatography–Mass Spectrometry (LC-MS)	Analyzes a wide range of metabolites of varying molecular weight. Detects both hydrophilic and hydrophobic compounds. Does not require derivatization. Better compound resolution than GC-MS.	LC-MS can be completed in a few minutes using short columns; but this can create suppression effects. These effects can also be caused by flow injection analysis (FIA)	Putri et al. [18] Lim et al. [44]
Ultra-High Performance Liquid Chromatography (UHPLC)	Higher signal-to-noise ratio, sensitivity, and specificity than NMR. Dependent on the detection device that is used (such as MS)	Fractionation requires greater pressure through columns of smaller particle sizes.	Nordström et al. [45] Zhang et al. [46]
Fourier-Transform Ion Cyclotron Resonance (FT-ICR)	Lower limit of detection and precise measurement. Simple sample preparation. Most sensitive method for detecting metabolites in complex mixtures. Can use either Electrospray ionization (ESI )or ApCl ionization.	Slower acquisition rates of the Fourier transform mass spectrometry (FTMS). Instrumentation is large and expensive.	Brown et al. [47] Ghaste et al. [48] Lim et al. [44]
Triple-Quadrupole Tandem MS (MS/MS)	Can be combined with either GC or LC. Reduces background noise stemming from solvent. Can be combined with Selection Reaction Monitoring (SRM) to further fragment ions and create a more sensitive platform.	Requires precursor ion selection before experiment; single-quadrupole mode must be used before triple-quadrupole. Total number of ions entering ion-trap is restricted, causes difficulty with small amounts of analyte in question (“ion suppression”)	Alder et al. [49] Weinmann et al. [50] Wu et al. [51]
